# Factors associated with 30-day and 1-year readmission among psychiatric inpatients in Beijing China: a retrospective, medical record-based analysis

**DOI:** 10.1186/s12888-020-02515-1

**Published:** 2020-03-11

**Authors:** Xueyan Han, Feng Jiang, Yilang Tang, Jack Needleman, Moning Guo, Yin Chen, Huixuan Zhou, Yuanli Liu

**Affiliations:** 1 School of public health, Chinese Academy of Medical Sciences and Peking Union Medical College, No.3 Dong Dan San Tiao, Dongcheng District, Beijing, China; 2 Department of Psychiatry and Behavioral Sciences, Emory University, 12 Executive Park Drive NE, Suite, Atlanta, GA 300 USA; 3 Atlanta VA Medical Center, 1670 Clairmont Road, Decatur, GA USA; 4 Department of Health Policy and Management, UCLA Fielding School of Public Health, 650 Charles Young Dr. S., 31-269 CHS Box, Los Angeles, CA 951772 USA; 5 Beijing Municipal Health Commission Information Centre, No. 277 Zhao Deng Yu Lu, Xicheng District, Beijing, China; 6 Peking University International Hospital, No. 29 Sheng Ming Yuan Lu, Haidian District, Beijing, China; 7 School of Sport Science, Beijing Sport University, No. 48 Xin Xi Lu, Haidian District, Beijing, China

**Keywords:** Psychiatric readmission, Psychiatric hospitals, China, Frequent readmissions, Comorbidities, Electroconvulsive therapy

## Abstract

**Background:**

Psychiatric readmissions negatively impact patients and their families while increasing healthcare costs. This study aimed at investigating factors associated with psychiatric readmissions within 30 days and 1 year of the index admissions and exploring the possibilities of monitoring and improving psychiatric care quality in China.

**Methods:**

Data on index admission, subsequent admission(s), clinical and hospital-related factors were extracted in the inpatient medical record database covering 10 secondary and tertiary psychiatric hospitals in Beijing, China. Logistic regressions were used to examine the associations between 30-day and 1-year readmissions plus frequent readmissions (≥3 times/year), and clinical variables as well as hospital characteristics.

**Results:**

The 30-day and 1-year psychiatric readmission rates were 16.69% (1289/7724) and 33.79% (2492/7374) respectively. 746/2492 patients (29.34%) were readmitted 3 times or more within a year (frequent readmissions). Factors significantly associated with the risk of both 30-day and 1-year readmission were residing in an urban area, having medical comorbidities, previous psychiatric admission(s), length of stay > 60 days in the index admission and being treated in tertiary hospitals (*p* < 0.001). Male patients were more likely to have frequent readmissions (OR 1.30, 95%CI 1.04–1.64). Receiving electroconvulsive therapy (ECT) was significantly associated with a lower risk of 30-day readmission (OR 0.72, 95%CI 0.56–0.91) and frequent readmissions (OR 0.60, 95%CI 0.40–0.91).

**Conclusion:**

More than 30% of the psychiatric inpatients were readmitted within 1 year. Urban residents, those with medical comorbidities and previous psychiatric admission(s) or a longer length of stay were more likely to be readmitted, and men are more likely to be frequently readmitted. ECT treatment may reduce the likelihood of 30-day readmission and frequent admissions. Targeted interventions should be designed and piloted to effectively monitor and reduce psychiatric readmissions.

## Background

According to the *China Mental Health Survey*, the lifetime prevalence of mental disorders was estimated to be 16.6% in China [1], indicating a significant disease burden. Psychiatric patients are prone to readmissions [2]: almost 1 in 7 psychiatric patients needed to be re-hospitalized within 30 days of discharge [3], and around 40% of the patients were readmitted 1 year after the index admissions [4]. Understanding the factors associated with readmission may provide important references to predict or prevent readmissions and/or frequent hospitalizations [5].

Although repeated hospitalizations for patients with psychiatric disorders may reflect the nature and course of the illnesses, socio-demographic or environmental factors, underlying inefficiencies during the hospitalization and in the pre- and post-discharge settings may also play a role [6]. Readmissions may be disruptive to patients and their families and place a strain on already limited healthcare resources. Moreover, repeated admissions could encourage dependency on inpatient services [7] and increase healthcare costs [5]. Therefore, readmission has long been recognized as a validated outcome measure for healthcare quality [8]. The commonly used timeframes to assess readmission were readmission within 30 days [3, 5, 7, 9] and 1 year [2, 7, 10]. As for the frequency of readmissions, Langdon et al. considered the patients that experienced three or more admissions a year to be “the revolving door” patients [11].

Previous studies have identified several factors associated with psychiatric readmissions, although some of these factors were found to be specific to settings and timeframes and the findings were somewhat mixed [5, 7]. Literature review showed that the following factors have been examined: 1) Socio-demographic factors: patients’ sex [4], age [11], marital status, insurance type, employment status, income level, care-giver information, living condition [12], education level and location of residency [5]; 2) Clinical factors: the previous service use [3], whether a patient was detained by law enforcement or was admitted involuntary [7], primary discharge diagnosis [13], illness severity (determined by various scales), various medical comorbidities [14], use of electroconvulsive therapy (ECT) [15], inpatient length of stay [16]; 3) Post-discharge factors: medication adherence [17], suicide attempts, family and social support, use of primary/community healthcare services; 4) Hospital characteristics: the hospital type, size [15] and location [9].

Most of the existing studies were conducted in developed countries, where the psychiatric care had been drastically reshaped after the de-institutionalization movement [4] and the expansion of community-based services. Few studies have been done in developing countries [10], where mental health services are vastly different in resource allocation and structure. For example, mental health services in China are mostly organized around psychiatric hospitals, which provide nearly 80% of mental health services (78.8% of all beds, and 78.4% of mental health workers) in the country [18, 19]. Therefore, it is of importance to study the pattern of psychiatric readmission and the associated factors in China.

China is currently facing a shortage in psychiatric beds and mental health professionals. For example, a recent study pointed out that there were 3.15 psychiatric beds per 10,000 population in China, nearly half of the rate in high-income countries (7.13 beds per 10,000 population) [20]. At the same time, the distribution of the already insufficient mental care resources is highly uneven. The community mental health services and mental health services in general hospitals are scarce [21]. Patients often tended to bypass the primary and secondary care and seek treatment directly in tertiary psychiatric hospitals [22], which are mainly located in urban areas of the more developed regions [23]. The lack of care coordination and psychiatric rehabilitation facilities also led to prolonged hospital stays (average psychiatric inpatient length of stay in China was 50–60 days in 2017 [24]).

Furthermore, China is a geographically vast country, and regional differences in service volume and clinical practice may exist. Take ECT treatment as an example, a survey of 45 psychiatric hospitals from 10 provinces in China, showed that the rates of ECT utilization among schizophrenia patients varied from 0.5% in Jiangsu province (eastern China) to 12.2% in Jilin province (northeast China) [25].

A few previous studies have focused on psychiatric readmission in patients in China. Zhou et al. (2014) identified previous hospitalizations to be significantly associated with 1-year readmission [2]. Zhang and Dai identified that being married, living with family members, low disease severity and good medication adherence were associated with a lower risk of readmission among 963 patients [26]. However, most of these studies were based on small sample sizes [27, 28] or based on patient records of a single hospital [29, 30].

To address this research gap, we designed this study, which aimed to investigate factors associated with 30-day and 1-year psychiatric readmissions as well as frequent readmissions (≥3 times/year). We utilized a regional, multi-center database to ensure a larger sample size and examine hospital-level variables. This study also utilized an all-payer database that included inpatient records regardless of their insurance types or payment methods, thus allowing us to include uninsured patients or patients covered by insurances other than the 3 main social health insurance schemes in China (namely the Urban employee basic medical insurance, Urban resident basic medical insurance and New cooperative medical scheme) [31].

## Methods

### Data source

The initial dataset was obtained from the Beijing Municipal Health Commission Information Centre (referred as the Information Centre). It contained data extracted from the face sheets of inpatient medical records [32] uploaded from 186 secondary and tertiary hospitals in Beijing city, China. The data used in this study was from the year 2015 to 2017. The databases covered 84.93% (186/219) of all the secondary and tertiary, public and private hospitals in Beijing [33], and included 10 (71.42%) out of 14 secondary and tertiary specialty psychiatric hospitals in the city [24].

**Fig. 1:**
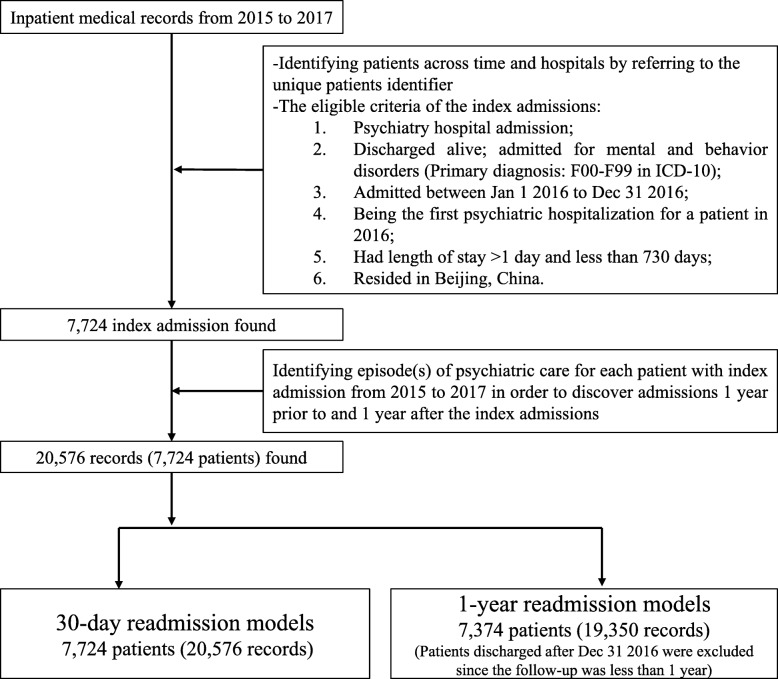
Flow chart for data cleansing and the identification of index admissions. Figure 1 illustrated the data cleansing and sample identification process of the study. It specified the eligibility criteria of the index admissions and the records included for both the 31-day and the 1-year psychiatric readmission models

The eligibility criteria of the index admissions and the data cleaning process were illustrated in Fig. 1. Each unique patient was identified using a patient identifier, which was generated and provided by the Information Centre. The patient identifier was a series of numbers unique to each patient and was used to recognize patients across time and different hospitals. This identifier contained no traceable personally identifiable information of the patients. Other variables used to filter eligible records were: hospital category code (to differentiate psychiatric hospitals from general hospitals), discharge destination code (to identify and exclude in-hospital deaths), primary diagnosis, date of admission and discharge, inpatient length of stay (LOS) and postal code of patients’ present address (to identify if the patients were residing in Beijing). Readmissions within 24 h of the last discharge were excluded as they were considered as planned admission or planned patient transfer [2]. Psychiatric inpatient stays for 24 h or less were not considered an index or readmissions since the patients were likely to be hospitalized for scheduled rehabilitation.

This project was approved by the Ethics Committee of Chinese Clinical Trial Registry (ChiECRCT-20,180,166). The informed consent of patients was waived.

### Study design

#### Model design

We constructed 3 multi-variate logistic models primarily: 1) the 30-day readmission model. The dependent variable was a binary variable indicating 30-day readmissions status (yes or no). All the eligible index admissions were included in this model; 2) the 365-day (1-year) readmission model. The dependent variable of this model was a binary variable indicating 365-day readmissions status (yes or no). The eligible index admissions with a discharge date after Dec 31, 2016 were excluded in this model. We applied this exclusion because we only had inpatient records up to Dec 31, 2017; 3) the frequent readmissions model. The dependent variable of this model was a binary variable with patients who had three or more psychiatric readmissions within 365 days of the index admissions versus patients with at least one and less than 3 readmissions within 365 days. The eligible index admissions with a discharge date after Dec 31, 2016 as well as eligible index admissions that were not followed by any readmission within 365 days after the index admissions were excluded. This model was designed to explore factors associated with repeated readmissions.

We used the same set of independent variables for these three models. We only included admissions with primary diagnoses of mental and behavioral disorders (ICD-10 code: F00-F99). We did not include admissions for non-psychiatric conditions within the timeframe.

#### Independent variables

We included a set of variables based on literature review and availability in our database. The variables included in this study were socio-demographic factors (sex [4], age, marital status, insurance type [2], patients’ residency (urban/rural) (based on their postal codes and the city planning of Beijing)); primary diagnostic categories (ICD 10 codes: depressive disorder (F32-F33), bipolar disorder (F31), schizophrenia and related disorder (F20-F29), substance use disorder (F10-F19), and other psychiatric disorders); number of medical comorbidities [14] (31 comorbidities defined in the AHRQ Elixhauser comorbidity index [14, 34, 35] were identified using the Stata module “ELIXHAUSER” and counted); treatment-related factors (use of electroconvulsive therapy (ECT) [15] (identified by the ICD-9-CM-3 code for ECT, 94.27)); previous admission(s) 1 year prior to the index admissions [5] (none, 1 times, 2 times, 3 times or more) (matched using patient identifier across hospitals and years); length of stay (days); as well as institutional factors [5, 15], including hospital level (tertiary or secondary) and hospital location (urban or rural). The comorbidities listed in the Elixhauser comorbidity index included cardiovascular conditions liver and renal diseases [36] (See Supplementary Table 1 in Additional File 1 for details). The Elixhauser comorbidity index has been used previously in risk-adjustment methodologies and predictive models [34].

### Statistical analyses

Descriptive analyses were conducted to show distributions of the independent variables in the entire sample and in the readmitted patients. Chi square analyses were applied to categorical variables and the Wilcoxon rank-sum test was applied to continuous variables to determine whether there were significant differences in groups with different readmission status.

Statistical inference was conducted using logistic regression. We used univariate logistic regressions first to examine the unadjusted effect between the different factors and the risk of readmission. We then used multi-variable logistic regressions on each dependent variable and generated estimates for all the independent variables. Unadjusted and adjusted odds ratios and 95% confidence intervals were reported. The pseudo R-square were reported for each model to show the general model performance. The variance inflation factors (VIF) for the models were also calculated to flag multi-collinearity. For model discrimination and calibration, the C-statistic was calculated from the receiver-operator characteristic curve to assess model discrimination, where a C-statistic of 0.50 or more indicates acceptable predictive power [37]. Hosmer-Lemeshow goodness-of-fit tests were applied and the calibration plots were generated for each model to assess model calibration [38].

To check the robustness of the results, we conducted sensitivity analyses to examine the same set of factors for index admissions for patients with schizophrenia and related disorders (F20-F29 as their primary diagnosis) and affective disorders (with F30-F39 as their primary diagnosis) respectively. All analyses were conducted using the Stata, version 15 (StataCorpLP, College Station, TX, USA). All *P*-values were two-sided and were considered significant at *p* < 0.05.

## Results

### Patient characteristics

Among the 7724 patients (index admissions) included in the analyses, 1289 patients (1289/7724, 16.69%) were readmitted within 30 days, and 2492/7374 (33.79%) were readmitted within 1 year after the index admissions in which 746/2492 patients (29.94%) were readmitted 3 or more times (Table 1). Among the 10 hospitals, 3 were tertiary hospitals and 5 were located in an urban area.

**Table 1:** Social-demographic and clinical characteristics of included patients

	30-day readmission (*N* = 7724)		1-year readmission (*N* = 7374)		
Characteristics (n (%))	All	Readmitted (*n* = 1289/7724)	All	Readmitted (*n* = 2492/7374)	Readmitted ≥3 times(*n* = 746/2492)
Gender: male	3708 (48.01)	713 (55.31)	3525 (47.8)	1318 (52.89)	465 (62.33)
Age (mean, SD)	46.71 (17.02)	54.71 (16.10)	46.27 (17.06)	50.50 (17.07)	58.05 (15.24)
Insurance*					
Urban employee	4350 (56.32)	979 (75.95)	4138 (56.12)	1707 (68.5)	629 (84.32)
Urban resident	859 (11.12)	82 (6.36)	795 (10.78)	242 (9.71)	42 (5.63)
New rural cooperative	633 (8.20)	49 (3.80)	600 (8.14)	116 (4.65)	10 (1.34)
Other insurances	918 (11.89)	110 (8.53)	946 (12.83)	189 (7.58)	19 (2.55)
Non-insured	964 (12.48)	69 (5.35)	895 (12.14)	238 (9.55)	46 (6.17)
Married	3777 (48.90)	416 (32.27)	3668 (49.74)	1020 (40.93)	228 (30.56)
Urban residents	5118 (66.26)	1023 (79.36)	4918 (66.69)	1883 (75.56)	646 (86.60)
Diagnoses					
Depressive disorders	1195 (15.47)	71 (5.51)	1192 (16.16)	234 (9.39)	35 (4.69)
Bipolar disorders	1635 (21.17)	123 (9.54)	1615 (21.9)	420 (16.85)	43 (5.76)
Schizophrenia and related disorders	3448 (44.64)	893 (69.28)	3169 (42.98)	1375 (55.18)	517 (69.30)
Substance use disorders	407 (5.27)	35 (2.72)	403 (5.47)	132 (5.30)	30 (4.02)
Other mental disorders	1039 (13.45)	167 (12.96)	995 (13.49)	331 (13.28)	121 (16.22)
Count of comorbidities^#^					
None	3268 (42.31)	234 (18.15)	3125 (43.60)	645 (25.88)	82 (10.99)
1	1759 (22.77)	287 (22.27)	1682 (22.81)	568 (22.79)	154 (20.64)
> 1	2697 (34.92)	768 (59.58)	2477 (33.59)	1279 (51.32)	510 (68.36)
Use of ECT	2065 (26.73)	119 (9.23)	2053 (27.84)	451 (18.10)	41 (5.50)
Previous hospitalization(s)					
None	5686 (73.61)	395 (30.64)	5550 (75.26)	1233 (49.48)	122 (16.35)
1 time	1054 (13.65)	258 (20.02)	878 (11.91)	430 (17.26)	92 (12.33)
2 times	544 (7.04)	334 (25.91)	509 (6.9)	421 (16.89)	226 (30.29)
≥ 3 times	440 (5.70)	302 (23.43)	437 (5.93)	408 (16.37)	306 (41.02)
Admission sources					
Emergency room	2528 (32.73)	172 (13.34)	2515 (34.11)	591 (23.72)	70 (9.38)
Outpatient	4914 (63.62)	1093 (84.79)	4663 (63.24)	1848 (74.16)	670 (89.81)
Other	282 (3.65)	24 (1.86)	196 (2.66)	53 (2.13)	6 (0.80)
Length of stay (mean, SD)	66.90 (78.70)	115.61 (75.21)	55.76 (55.93)	79.83 (63.43)	101.33 (50.48)
Hospital type					
Tertiary hospitals (vs secondary hospitals)	6438 (83.35)	1119 (86.81)	6346 (86.06)	2135 (85.67)	684 (91.69)
Urban hospitals (vs rural hospitals)	4591 (59.44)	298 (23.12)	4490 (60.89)	1084 (43.50)	146 (19.57)

Notes: * The full titles of the first 3 insurance types are Urban employee basic medical insurance, Urban resident basic medical insurance and New cooperative medical insurance. The same goes for other tables

^#^ 31 comorbidities defined by the AHRQ Elixhauser Comorbidities Index were identified by the Stata module “ELIXHAUSER” and then counted. The same goes for other tables

Regarding the sociodemographic factors, 51.99% of the included patients were female, the mean age was 46.71 years old (standard deviation: 17.02 years), nearly half of the patients were married (48.90%), and 87.52% had health insurance coverage. Clinically, more than one fourth (26.39%) had previous admission(s) within a year prior to the index admissions. The largest diagnosis group was schizophrenia and related disorders (44.64%), followed by bipolar disorders (31.17%). More than half of the sample (57.69%) had at least 1 medical comorbidity listed in the Elixhauser comorbidity index. The mean inpatient length of stay (LOS) was 66.90 days, with a great range of variation (median: 36 days; interquartile range: 60 days; standard deviation: 78.70 days). Details about the basic features of the sample can be found in the Additional File 1 (Supplementary Table 2–4).

### Thirty-day readmissions and associated factors

For unadjusted analyses, sex, age, marital status and primary diagnosis group were all significantly associated with readmission risk but none of them were statistically significant in the multi-variable regression model. The variables that were significantly associated with the risk of 30-day readmission including an urban residency, medical comorbidities, prior psychiatry hospitalizations in the previous year, longer inpatient length of stay (> 60 days), as well as being treated in tertiary hospitals (*p* < 0.001) (Table 2). The use of ECT was significantly associated with a low risk of 30-day readmission. The Pseudo-R^2^ of this model was 0.35 and C-statistics was 0.87, indicating acceptable predicting power. Detailed test results of model discrimination and calibration can be found in the Additional File 1 (Supplementary Table 8).

**Table 2:** Logistic regression of analysis of readmissions in 30-days and 1 year

	30-day readmission model (N = 7724)		1-year readmission model (N = 7374))	
	Univariate models	Multivariate model	Univariate models	Multivariate model
Male (ref. female)	1.42 (1.26, 1.60)**	0.95 (0.81, 1.11)	1.36 (1.24, 1.5)**	1 (0.89, 1.12)
Age (ref. (0–40])				
(40,65]	2.63 (2.25, 3.06)**	0.84 (0.68, 1.04)	1.72 (1.54, 1.91)**	0.8 (0.69, 0.92)**
> 65	4.71 (3.90, 5.68)**	0.92 (0.69, 1.21)	2.72 (2.33, 3.17)**	0.69 (0.55, 0.87)**
Insurance type (ref. no insurance)				
Urban employee	3.76 (2.91, 4.86)**	1.19 (0.88, 1.6)	2.81 (2.37, 3.34)**	1.42 (1.17, 1.72)**
Urban resident	1.37 (0.98, 1.91)	0.75 (0.51, 1.09)	1.75 (1.41, 2.18)**	1.22 (0.96, 1.56)
New rural cooperative	1.08 (0.74, 1.59)	1.12 (0.74, 1.71)	0.96 (0.74, 1.24)	0.99 (0.75, 1.31)
Other insurances	1.77 (1.29, 2.42)**	1.03 (0.71, 1.5)	1.45 (1.17, 1.8)**	0.86 (0.76, 0.99)*
Married (ref. not married)	0.44 (0.38, 0.50)**	0.86 (0.72, 1.02)	0.58 (0.53, 0.64)**	0.86 (0.76, 0.97)**
Urban residents (ref. Suburb)	2.19 (1.90, 2.54)**	1.4 (1.15, 1.7)**	1.88 (1.69, 2.1)**	1.41 (1.23, 1.61)**
Diagnosis (ref. depressive disorders)				
Bipolar disorders	1.29 (0.95, 1.74)	1.1 (0.79, 1.54)	1.44 (1.2, 1.72)**	1.35 (1.11, 1.65)**
Schizophrenia and related disorders	5.53 (4.30, 7.12)**	1.44 (1.06, 1.94)*	3.14 (2.68, 3.68)**	1.35 (1.12, 1.63)**
Substance use disorders	1.49 (0.98, 2.27)	0.64 (0.4, 1.04)	1.99 (1.55, 2.57)**	1.14 (0.85, 1.53)
Other mental disorders	3.03 (2.27, 4.06)**	1.3 (0.93, 1.81)	2.04 (1.68, 2.48)**	1.18 (0.94, 1.47)
Count of comorbidities (ref. none)				
1	2.53 (2.10, 3.04)**	1.67 (1.34, 2.09)**	2.03 (1.78, 2.32)**	1.62 (1.39, 1.89)**
> 1	5.16 (4.41, 6.04)**	2.17 (1.75, 2.7)**	4.25 (3.78, 4.78)**	2.63 (2.25, 3.07)**
Use of ECT (ref. no ECT)	0.23 (0.19, 0.29)**	0.72 (0.56, 0.91)**	0.45 (0.4, 0.51)**	0.91 (0.79, 1.05)
Psychiatric hospitalizations 1 year prior to index admission (ref. none)				
1	4.34 (3.65, 5.17)**	1.91 (1.55, 2.36)**	3.36 (2.9, 3.89)**	1.96 (1.66, 2.31)**
2	21.30 (17.43, 26.04)**	4.87 (3.8, 6.24)**	16.75 (13.2, 21.26)**	6.19 (4.76, 8.05)**
≥ 3 times	28.31 (23.39, 36.74)**	5.12 (3.87, 6.78)**	49.26 (33.62, 72.17)**	15.84 (10.58, 23.72)**
Admission sources (ref. emergency room)				
Outpatient	3.92 (3.31, 4.64)**	1.13 (0.92, 1.39)	2.14 (1.92, 2.38)**	1.07 (0.94, 1.22)
Other	1.27 (0.82, 1.99)	0.53 (0.31, 0.9)*	1.21 (0.87, 1.68)	0.85 (0.57, 1.27)
Length of stay (ref. ≤20 days)				
(20,30]	0.45 (0.33, 0.62)**	0.45 (0.33, 0.62)**	0.9 (0.76, 1.06)	0.87 (0.73, 1.03)
(30,40]	0.52 (0.37, 0.73)**	0.46 (0.32, 0.66)**	0.89 (0.74, 1.07)	0.8 (0.66, 0.98)*
(40,60]	0.75 (0.56, 1.02)	0.53 (0.38, 0.74)**	1.13 (0.94, 1.35)	0.89 (0.73, 1.08)
(60,100]	4.35 (3.47, 5.46)**	1.6 (1.21, 2.13)**	3.12 (2.63, 3.71)**	1.39 (1.13, 1.72)**
> 100	10.40 (8.47, 12.77)**	3.27 (2.46, 4.35)**	7.96 (6.71, 9.45)**	2.29 (1.82, 2.89)**
Hospital type				
Tertiary hospitals (vs secondary hospitals)	1.38 (1.16, 1.64)**	2.61 (2.04, 3.35)**	0.95 (0.83, 1.09)	1.38 (1.14, 1.67)**
Urban hospitals (vs rural; hospitals)	0.15 (0.13, 0.17)**	0.54 (0.44, 0.66)**	0.33 (0.3, 0.37)**	0.75 (0.65, 0.87)**

Notes: * *p* < 0.05; ** *p* < 0.001; Number in the Table: Odds ratio (95% confidence interval)

### One-year readmission and associated factors

We found being married was significantly associated with a low risk of 1-year readmission risk (*p* < 0.05). Compared to patients with depressive disorders, patients with bipolar disorders and schizophrenia and related disorders were significantly associated with risk of 1-year readmission (*p* < 0.001). Full results are displayed in Table 2. ECT was significantly associated with a low risk of readmission in the univariate model, but the significance disappeared in the multi-variable analysis. The Pseudo-R^2^ of this model was 0.23 and C-statistics was 0.78.

### Factors associated with 1-year frequent readmissions

Among 2492 patients who were readmitted at least once within a year after the index admissions, nearly one third (29.94%) were readmitted for 3 or more times (frequent readmission). Compared with those with 1 or 2 readmissions, being male, residing in an urban area, having medical comorbidities, previous hospitalizations and being treated in a tertiary hospital were significantly associated with risk of frequent readmissions (Table 3). The use of ECT was associated with a lower risk of frequent readmission (OR: 0.60, 95%CI: 0.40–0.91). The Pseudo-R^2^ was 0.31 and C-statistics was 0.86 for this model.

**Table 3:** Logistic regression of 1-year frequent readmission (≥3 times/1 year) (N = 2492)

	Frequent readmission in 1 year (746/2492)	
	Univariate models	Multivariate model
Male (ref. female)	1.73 (1.45, 2.06)**	1.3 (1.04, 1.64)*
Age (ref. (0–40])		
(40,65]	3.19 (2.5, 4.07)**	1.06 (0.77, 1.46)
> 65	6.39 (4.82, 8.48)**	1.26 (0.84, 1.89)
Insurance type (ref. no insurance)		
Urban employee	5.22 (3.22, 8.47)**	1.69 (0.96, 2.96)
Urban resident	1.88 (1.05, 3.35)	1.2 (0.61, 2.34)
New rural cooperative	0.84 (0.38, 1.88)	1.3 (0.53, 3.16)
Other insurances	2.14 (1.21, 3.8)	0.92 (0.47, 1.81)
Married (ref. not married)	0.53 (0.44, 0.64)**	0.89 (0.68, 1.15)
Urban residents (ref. Suburb)	2.66 (2.1, 3.36)**	1.45 (1.07, 1.95)*
Diagnosis (ref. Depressive disorders)		
Bipolar disorders	0.65 (0.4, 1.05)	0.47 (0.27, 0.82)**
Schizophrenia and related disorders	3.43 (2.35, 4.99)**	0.78 (0.49, 1.26)
Substance use disorders	1.67 (0.97, 2.88)	0.81 (0.43, 1.54)
Other mental disorders	3.28 (2.15, 5)**	1.2 (0.72, 2.03)
Count of comorbidities (ref. none)		
1	2.55 (1.9, 3.44)**	1.59 (1.11, 2.27)*
> 1	4.55 (3.52, 5.89)**	1.95 (1.4, 2.71)**
Use of ECT (ref. no ECT)	0.19 (0.14, 0.26)**	0.6 (0.4, 0.91)*
Psychiatric hospitalizations 1 year prior to index admission (ref. none)		
1	2.48 (1.84, 3.34)**	1.66 (1.19, 2.32)**
2	10.55 (8.08, 13.79)**	4.79 (3.45, 6.66)**
≥ 3 times	27.32 (20.41, 36.58)**	10.02 (7.05, 14.24)**
Admission sources (ref. emergency room)		
Outpatient	4.23 (3.24, 5.53)	1.29 (0.91, 1.82)
Other	0.95 (0.39, 2.3)	0.93 (0.32, 2.75)
Length of stay (ref. ≤20 days)		
(20, 30]	0.63 (0.38, 1.05)	0.62 (0.36, 1.08)
(30, 40]	0.96 (0.57, 1.62)	0.84 (0.47, 1.49)
(40, 60]	1.66 (1.06, 2.58)*	1.3 (0.79, 2.14)
(60, 100]	8.13 (5.65, 11.68)**	2.48 (1.56, 3.95)**
> 100	6.01 (4.26, 8.47)**	1.26 (0.79, 2.01)
Hospital type		
Tertiary hospitals (vs secondary hospitals)	2.24 (1.68, 2.99)**	2.3 (1.51, 3.51)**
Urban hospitals (vs rural hospitals)	0.21 (0.17, 0.26)**	0.86 (0.62, 1.19)

Notes: * *p* < 0.05; ** *p* < 0.001; Number in the Table: Odds ratio (95% confidence interval)

### Sensitivity analyses

The results for models in the sensitivity analyses as well as the post-regression diagnostics tests were presented in the Additional File 1 (Supplementary Table 5–9). The models including patients with schizophrenia and related disorders basically showed consistent results with the full models that included all the patients. The association between the use of ECT and low readmission risk was found in the schizophrenia models, but the association was not significant in the affective disorder models, regardless of readmission interval or frequency.

## Discussion

### Main findings

This study investigated the factors associated with risk of psychiatric readmission within 30 days and 1 year after the index admissions in the regional all-payer database of Beijing, China. The 30-day and 1-year psychiatric readmission rates were 16.69 and 33.79% respectively and 746 patients out of 7374 patients (10.12%) were re-admitted three times or more within a year. Several factors were significantly associated with a high risk of 30-day and 1-year readmission, including an urban residency, having more medical comorbidities and previous psychiatric admission(s), a length of stay of > 60 days in the index admission as well as being treated in tertiary hospitals. The use of ECT was significantly associated with a lower risk of 30-day readmissions and frequent readmissions in the whole sample and the schizophrenia subgroup.

### Psychiatric readmission rates

The 30-day readmission rate (16.69%) in this study was consistent with some studies conducted in other countries and settings [7, 9]; nevertheless, the 30-day readmission rate had large variations from previous reports. A report published by the Organization for Economic Cooperation and Development suggested that the 30-day readmission rate for schizophrenia varied from 5 to 20% among 20 countries [39] and the 30-day readmission rate was 4.5% (1598/37,796) in a Korean sample [8]. The 1-year readmission rate (33.79%) in our study is similar to that reported in other studies [10, 40] except the one reported by Zhou el al. (2014) which reported the 1-year readmission rate as 13.8% (476/3455) in one hospital in Guangzhou city [2]. There are several possible explanations for the different rates of readmission, including differences in patient populations, length of stay, discharge criteria and insurance coverage. The inclusion/exclusion criteria are also different from one study to another. Our study was able to track the patients even if the patients were admitted to a different hospital or changed payment methods between admissions. This study also adopted more strict inclusion criteria. For instance, current residency in Beijing was considered one of the eligibility criteria so that non-residents would not inflate the denominator and cause an under-estimation of the readmission rate.

### Patient characteristics

We found that being male, with a younger age and being unmarried were significantly associated with a higher risk of readmission. Such findings were consistent with the results of several other studies according to a systematic review published in 2016 [5]. This may reflect the differences in the symptom patterns and illness courses among patients with varied socio-demographic backgrounds [41]. An urban residency was found to be a risk factor for short-term and frequent readmissions, which was consistent with the results of Tami et al. [12] but since this factor had rarely been included, the mechanism behind such connection is unclear [5]. It may be confounded by the difference in income, education status, care-giving method, insurance coverage, family support and neighborhood environment between the urban and rural residency. It may reflect a difference in service accessibility and availability as urban residents tend to have better resources and access to psychiatric care.

With respect to the LOS, we found the LOS in China (mean: 66.90 days) was considerably longer than that in many developed countries (mostly less than 40 days) [2, 4, 42]. This reflects the reality that psychiatric hospitalization remains a primary source of treatment for many patients and that there is an urgent need to expand community-based mental health services [2, 43, 44].

Our findings provide an opportunity to further examine the effects of different LOS on risk of readmission. Compared with patients with LOS less than 20 days, patients with LOS between 20 to 60 days had a lower readmission risk while the ones with LOS > 60 days had a higher risk. This may suggest that the optimal LOS is between 20 and 60 days in the study population although further studies are needed in this area. It is reasonable to assume that for the patients with LOS longer than 60 days, they were more likely to have a more severe disorder, and they were more likely to be treatment-resistant and to have limited family or social support.

### The use of electroconvulsive therapy (ECT)

The study found that the use of ECT was significantly associated with a low risk of the 30-day and frequent readmissions in the entire sample and among the patients with schizophrenia and related disorders. Among the patients with affective disorders (depressive and bipolar disorders), a trend of association was found between ECT treatment and a low risk of readmission but the association was not statistically significant.

ECT is widely used in China and unlike in United States, where ECT is predominantly used for patients with major depressive disorder, it is used in patients with numerous other psychiatric disorders, with schizophrenia and mood disorders being the two most common indications [45, 46].

Our findings about the association between ECT and readmission risk are important and intriguing. It is important because we found that ECT was associated with a lower risk of readmission in the whole sample including patients from 10 hospitals, and in the sample with schizophrenia and related disorders. It is intriguing because the association was not significant among patients with affective disorders, which is inconsistent with the findings by Slade et al. (2017) [15], who found the use of ECT was significantly associated with reduction of 30-day readmission risk in 162,691 US patients with severe affective disorders from 9 states [15]. Our findings are also different from another study in China (Ma et al. (2019)), which analyzed discharge data in one psychiatric hospital in Guangzhou city, and found no significant association between the use of ECT and the risk of 6-month readmissions [46].

These discrepancies may due to different inclusion and diagnostic criteria, varied ECT utilization rates (our study: 27%; Ma 2019: 17.8%; Slade 2017: 1.5%) and even different treatment parameters in different studies. Other factors, such as other treatments (including medications) during the index admissions, and the number of ECT treatments, may also play a role. Although the current finding of this study may suggest that ECT is an effective treatment in reducing the risk of readmissions in the study population, it is also possible that patients or their families are avoidant of future admissions, due to actual or perceived side effects or other reasons (such as stigma or fear) related to ECT treatments. Unfortunately, we do not have the relevant data to further understand the mechanism behind the findings.

Clearly, the association between ECT and readmission rate warrants further research. Since the association between ECT use and the risk of readmission can be disorder and study setting specific (rate of utilization varies), future studies may need to include more details, such as data on the patients’ symptoms, medication use, treatment parameters, adverse events during the procedure, etc. [47].

### Hospital characteristics

We found that index admission in tertiary hospitals was significantly associated with 30-day and frequent readmissions in this study. This could be explained by the fact that patients treated in the tertiary hospitals tended to be more severe and treatment-resistant, and such severity was not fully adjusted for using the existing variables in the multi-variate model. Previous studies using data from the Centre of Medicare and Medicaid Services (CMS) in United States had identified that larger, urban, academic facilities tended to have slightly higher risk-standardized readmission rates [48, 49]. In this study, tertiary hospitals in China normally had more hospital beds and higher service volumes so that the finding of this research was in line with the results of previous studies. On the other hand, compared with admission at rural hospitals, admission at urban hospitals was associated with a low risk of readmission in this study, this may suggest that urban hospitals may provide better services or are better at discharge planning or service coordination. Further studies may try to include more psychiatric hospitals located in more regions of China, so that more institutional characteristics, like staffing levels [50] and geographical variables can be incorporated into the analyses.

### Strengths and limitations

One of the strengths of this study is that we utilized the regional all-payer medical record database from 2015 to 2017, providing evidences on the 30-day, 1-year and frequent readmissions of psychiatric patients in Beijing. The sample is very large (> 7000) and multiple hospitals in different levels (tertiary versus secondary) and different setting (urban versus rural) were included. The regional multi-center database in this study is better than data from a single hospital, as it may detect readmissions across hospitals and institutional factors can be examined as well.

Another strength is the rigorousness of inclusion/exclusion criteria and the inclusion of ECT treatment in the analysis. For example, we excluded patients with unusual LOS (< 24 h) and patients who were non-Beijing residents to reduce the bias introduced by patients admitted for rehabilitation purposes or patients travelled to Beijing for treatments.

A few limitations also need to be acknowledged. Firstly, since we only used data from the face sheets of discharge medical records, we were unable to include several potentially important factors. For example, we were unable to include demographic factors such as employment, income, living situation and education levels; clinical factors such as the severity of the psychiatric symptoms, the status of voluntarily versus involuntary admission, patients’ response to medications; as well as post-discharge factors like medication adherence, support system etc. While this study proved that face sheets of medical records can be used as a cost-effective way to examine factors associated with readmission risk, further prospective studies are needed to replicate our findings and explore the mechanism behind the associations between the identified factors and the readmission risk. Due to the limitation of our dataset, some of our findings, especially the findings regarding ECT treatment, should be interpreted with caution, since we only used ECT treatment as a categorical variable (yes or no). Future studies need to include treatment-related factors, including number of treatments, legal status, treatment parameters (such as electrode placement, charge, etc.) and adverse events.

Secondly, we did not have access to the outpatient records of those patients thus we were unable to examine the outpatient service utilization after the index admissions. We also did not have information on the community care availability and intensity [26] so we were unable to examine their effects on the risk of readmission or incorporate these factors in the risk-adjustment models. We also did not include patient readmissions due to physical illnesses so the psychiatric readmission rate estimated in this study may be lower than the all-cause readmission rates among psychiatric inpatients [51].

Thirdly, since this study used data from Beijing, the capital city of China and it is considered as a regional and even national medical center with better resources, the results of this study may not be generalizable to other parts of the country, especially less developed regions. A national database or a national represented sample of psychiatric patients would be more ideal data sources for future studies.

## Conclusions

This study estimated that the rate of 30-day and 1-year psychiatric readmission was 16.69 and 33.79% respectively; and 10.12% were considered as frequent readmissions (≥3 admissions within 12 months after discharge). Those who resided in an urban area, with medical comorbidities, previous psychiatric admissions, and length of stay of > 60 days in the index admissions were more likely to have psychiatric readmissions. Receiving ECT treatment may be associated with a lower risk of readmission.

Further studies might consider exploring the clinical and epidemiological mechanisms behind the risk and protective factors for readmission or designing interventions at hospital and community levels to reduce psychiatric readmission in China.

## Supplementary information

**Additional File 1.** Results of supplementary analyses and the list of comorbidities included in the Elixhauser Comorbidity Index (ECI). Supplementary Table 1 listed the comorbidities of ECI. Supplementary Table 2–4 showed the results of the supplementary descriptive and univariate analyses. Supplementary Table 5–7 presented the results of the sensitivity analyses. Supplementary Table 8–9 showed the results for post-regression diagnostics and calibration plots of the primary and sensitivity analyses models.

## Data Availability

The data used in this study were obtained from the inpatient medical record (face sheets) database of Beijing Municipal Health Commission Information Centre and was not publicly available. Contact information the Information Centre can be found on http://www.phic.org.cn/tjsj/.

## Abbreviations

AHRQ: Agency for Healthcare Research & Quality (United States)
CI: Confidence interval
ECT: electroconvulsive therapy
ICD: International Classification of Diseases
LOS: Length of stay
OR: Odds ratio
