# Supplementary material for: Factors associated with 30-day and 1-year readmission among psychiatric inpatients in Beijing China: a retrospective, medical record-based analysis
Source: BMC Psychiatry. 2020 Mar 11;20:113. doi: 10.1186/s12888-020-02515-1 (PMC7065326; doi:10.1186/s12888-020-02515-1)
Supplement: Supplementary file 1 — Additional File 1. Results of supplementary analyses and the list of comorbidities included in the Elixhauser Comorbidity Index (ECI). Supplementary Table 1 listed the comorbidities of ECI. Supplementary Table 2–4 showed the results of the supplementary descriptive and univariate analyses. Supplementary Table 5–7 presented the results of the sensitivity analyses. Supplementary Table 8–9 showed the results for post-regression diagnostics and calibration plots of the primary and sensitivity analyses models. [file 12888_2020_2515_MOESM1_ESM.docx]

Additional file for “**Factors associated with 30-day and 1-year readmission among psychiatric inpatients in Beijing China: A retrospective, medical record-based analysis**”

1. AHRQ Elixhauser comorbidity index

Supplementary table 1 showed the 31 comorbidities defined in the “ELIXHAUSER: Stata module to calculate Elixhauser index of comorbidity”. This module was created based on “Elixhauser A, Steiner C, Harris DR, Coffey RM: Comorbidity Measures for Use with Administrative Data. MED CARE 1998, 36(1):8-27” and can be found at <https://ideas.repec.org/c/boc/bocode/s458077.html>. This module can be cited as “Vicki Stagg, 2015. "ELIXHAUSER: Stata module to calculate Elixhauser index of comorbidity," Statistical Software Components S458077, Boston College Department of Economics”.

This study used this Stata module to identify patient co-morbidities in the secondary diagnosis codes of the patient records. The secondary diagnoses were coded in ICD-10.

The count of comorbidity=0 would mean that the patients had no comorbidity listed below.

| Supplementary table 1. Elixhauser comorbidities counted in this study | |
| --- | --- |
| No. | Elixhauser comorbidities |
| 1 | Congestive Heart Failure |
| 2 | Valvular disease |
| 3 | Cardiac Arrhythmias |
| 4 | Pulmonary circulation disorders |
| 5 | Peripheral vascular disease |
| 6 | Hypertension (uncomplicated) |
| 7 | Hypertension (complicated) |
| 8 | Paralysis |
| 9 | Other neurological disorders |
| 10 | Chronic pulmonary disease |
| 11 | Diabetes without chronic complications |
| 12 | Diabetes with chronic complications |
| 13 | Hypothyroidism |
| 14 | Renal failure |
| 15 | Liver disease |
| 16 | Chronic peptic ulcer disease |
| 17 | HIV and AIDS (Acquired immune deficiency syndrome) |
| 18 | Lymphoma |
| 19 | Metastatic cancer |
| 20 | Solid tumor without metastasis |
| 21 | Rheumatoid arthritis/ collagen vascular diseases |
| 22 | Coagulation deficiency |
| 23 | Obesity |
| 24 | Weight loss |
| 25 | Fluid and electrolyte disorders |
| 26 | Blood loss anemia |
| 27 | Deficiency anemias |
| 28 | Alcohol abuse |
| 29 | Drug abuse |
| 30 | Psychoses |
| 31 | Depression |

2. The supplementary descriptive analyses

In the main text, the univariate analyses were all logistic regressions with each independent variable being grouped into a categorical variable. This design was intended to compare the results of the univariate analysis and the multi-variate analysis of a same independent variable side by side, so that it would show the effect of adjusting for other included variables in the multiple regression model. The univariate logistic regression also showed the OR value for each independent variable instead of just indicating that there were differences among the groups.

However, this step of data transformation can lead to an unnecessary loss of information for the continuous variables and the basic hypothesis testing for all the independent variables could be informative. The Chi square analyses were applied to categorical variables and the Wilcoxon rank-sum test was applied to continuous variables. We presented Supplementary table 2-4 to show the results of the supplementary descriptive and univariate analyses.

| Supplementary table 2. Characteristics of the index admissions (for the 30-day psychiatric readmission analyses) | | | | |
| --- | --- | --- | --- | --- |
| Characteristics | All (N=7,724) | Readmitted in 30 days (n=1289/7724) | Not readmitted in 30 days (n=6,435/7724) | Statistics |
| Gender: male | 3708(48.01) | 713(55.31) | 2995(46.54) | χ2(1) = 33.10, P < 0.001 |
| Age (mean, SD) | 46.71(17.02) | 54.71(16.10) | 45.11(16.74) | z = -18.74, P < 0.001 |
| Insurance* |  |  |  | χ2(4) = 251.45, P < 0.001 |
| Urban employee | 4350(56.32) | 979(75.95) | 3371(52.39) |  |
| Urban resident | 859(11.12) | 82(6.36) | 777(12.07) |  |
| New rural cooperative | 633(8.20) | 49(3.80) | 584(9.08) |  |
| Other insurances | 964(12.48) | 69(5.35) | 895(13.91) |  |
| Non-insured | 918(11.89) | 110(8.53) | 808(12.56) |  |
| Married | 3777(48.90) | 416(32.27) | 3361(52.23) | χ2(1) = 171.17, P < 0.001 |
| Urban residents | 5118(66.26) | 1032(79.36) | 4095(63.64) | χ2(1) = 118.82, P < 0.001 |
| Diagnoses |  |  |  | χ2(4) = 427.89, P < 0.001 |
| Depressive disorders | 1195(15.47) | 71(5.51) | 1124(17.47) |  |
| Bipolar disorders | 1635(21.17) | 123(9.54) | 1512(23.50) |  |
| Schizophrenia and related disorders | 3448(44.64) | 893(69.28) | 2555(39.70) |  |
| Substance use disorder | 407(5.27) | 35(2.72) | 372(5.78) |  |
| Other mental disorders | 1039(13.45) | 167(12.96) | 872(13.55) |  |
| Count of comorbidities^#^ |  |  |  | χ2(2) = 483.10, P < 0.001 |
| None | 3268(42.31) | 234(18.15) | 3034(47.15) |  |
| 1 | 1759(22.77) | 287(22.27) | 1472(22.87) |  |
| >1 | 2697(34.92) | 768(59.58) | 1929(29.98) |  |
| ECT | 2065(26.73) | 119(9.23) | 1946(30.24) | χ2(1) = 241.99, P < 0.001 |
| Previous hospitalization |  |  |  | χ2(3) = 2100, P < 0.001 |
| None | 5686(73.61) | 395(30.64) | 5291(82.22) |  |
| 1 time | 1054(13.65) | 258(20.02) | 796(12.37) |  |
| 2 times | 544(7.04) | 334(25.91) | 210(3.26) |  |
| >3 times | 440(5.70) | 302(23.43) | 138(2.14) |  |
| Admission sources |  |  |  | χ2(2) = 300.25, P < 0.001 |
| Emergency room | 2528(32.73) | 172(13.34) | 2356(36.61) |  |
| Outpatient | 4914(63.62) | 1093(84.79) | 3821(59.38) |  |
| Other | 282(3.65) | 24(1.86) | 258(4.01) |  |
| Length of stay (mean, SD) | 66.90(78.70) | 115.61(75.21) | 57.14(75.71) | z = -30.73, P < 0.001 |
| Hospital info |  |  |  |  |
| Tertiary hospital vs secondary hospitals | 6438(83.35) | 1119(86.81) | 5319(82.66) | χ2(1) = 13.35, P < 0.001 |
| Urban hospital vs rural hospitals | 4591(59.44) | 298(23.12) | 4293(66.71) | χ2(1) = 846.53, P < 0.001 |
| Notes: * The full expression of the first 3 insurance types are Urban employee basic medical insurance, Urban resident basic medical insurance and New cooperative medical scheme. The same goes for other tables.  ^#^ 31 comorbidities defined by the AHRQ Elixhauser Comorbidities Index were identified by the Stata module “ELIXHAUSER” and then counted. The same goes for other tables. | | | | |

| Supplementary table 3. Characteristics of the index admissions (for the 365-day psychiatric readmission analyses) | | | | |
| --- | --- | --- | --- | --- |
| Characteristics | All (n=7374) | Readmitted in 365 days (n=2492/7374) | Not readmitted in 365 days (n=4882/7724) | Statistics |
| Gender: male | 3525(47.8) | 1318(52.89) | 2207(45.21) | χ2(1) = 39.02, P < 0.001 |
| Age (mean, SD) | 46.27(17.06) | 50.50(17.07) | 44.11(16.65) | z = -15.03, P < 0.001 |
| Insurance* |  |  |  | χ2(4) = 264.39, P < 0.001 |
| Urban employee | 4138(56.12) | 1707(68.50) | 2431(49.80) |  |
| Urban resident | 795(10.78) | 242(9.71) | 553(11.33) |  |
| New rural cooperative | 600(8.14) | 116(4.65) | 484(9.91) |  |
| Other insurances | 946(12.83) | 189(7.58) | 757(15.51) |  |
| Non-insured | 895(12.14) | 238(9.55) | 657(13.46) |  |
| Married | 3668(49.74) | 1020(40.93) | 2648(54.24) | χ2(1) = 116.90, P < 0.001 |
| Urban residents | 4918(66.69) | 1883(75.56) | 3035(62.17) | χ2(1) = 133.26, P < 0.001 |
| Diagnoses |  |  |  | χ2(4) = 281.37, P < 0.001 |
| Depressive disorder | 1192(16.16) | 234(9.39) | 958(19.62) |  |
| Bipolar disorder | 1615(21.9) | 420(16.85) | 1195(24.48) |  |
| Schizophrenia and related disorders | 3169(42.98) | 1375(55.18) | 1794(36.75) |  |
| Substance use disorder | 403(5.47) | 132(5.30) | 271(5.55) |  |
| Other mental disorders | 995(13.49) | 331(13.28) | 664(13.60) |  |
| Count of comorbidities^#^ |  |  |  | χ2(2) = 623.35, P < 0.001 |
| None | 3125(43.60) | 645(25.88) | 2570(52.64) |  |
| 1 | 1682(22.81) | 568(22.79) | 1114(22.82) |  |
| >1 | 2477(33.59) | 1279(51.32) | 1198(24.54) |  |
| ECT | 2053(27.84) | 451(18.1) | 1602(32.81) | χ2(1) = 177.86, P < 0.001 |
| Previous hospitalization |  |  |  | χ2(3) = 1700, P < 0.001 |
| None | 5550(75.26) | 1233(49.48) | 4317(88.43) |  |
| 1 time | 878(11.91) | 430(17.26) | 448(9.18) |  |
| 2 times | 509(6.9) | 421(16.89) | 88(1.80) |  |
| >3 times | 437(5.93) | 408(16.37) | 29(0.59) |  |
| Admission sources |  |  |  | χ2(2) = 194.14, P < 0.001 |
| Emergency room | 2515(34.11) | 591(23.72) | 1924(39.41) |  |
| Outpatient | 4663(63.24) | 1848(74.16) | 2815(57.66) |  |
| Other | 196(2.66) | 53(2.13) | 143(2.93) |  |
| Length of stay (mean, SD) | 55.76(55.93) | 79.83(63.43) | 43.48(47.18) | z = -26.40, P < 0.001 |
| Hospital info |  |  |  |  |
| Tertiary hospitals vs secondary hospitals | 6346(86.06) | 2135(85.67) | 4211(86.26) | χ2(1) = 0.46, P = 0.495 |
| Urban hospital vs rural admissions | 4490(60.89) | 1084(43.50) | 3406(69.77) | χ2(1) = 478.01, P < 0.001 |

| Supplementary table 4. Characteristics of the index admissions (for the frequent psychiatric readmission analyses) | | | | |
| --- | --- | --- | --- | --- |
| Characteristics | Patients got readmitted in 365 days (n=2492) | Readmitted ≥3 times in 365 days (n=746/2492) | Readmitted <2 times in 365 days (n=1746/2492) | Statistics |
| Gender: male | 1318(52.89) | 465(62.33) | 853(48.85) | χ2(1) = 38.11, P < 0.001 |
| Age (mean, SD) | 50.50(17.07) | 58.05(15.24) | 47.27(16.8) | z = -14.51, P < 0.001 |
| Insurance* |  |  |  | χ2(4) = 130.67, P < 0.001 |
| Urban employee | 1707(68.5) | 629(84.32) | 1078(61.74) |  |
| Urban resident | 242(9.71) | 42(5.63) | 200(11.45) |  |
| New rural cooperative | 116(4.65) | 10(1.34) | 106(6.07) |  |
| Other insurances | 189(7.58) | 19(2.55) | 170(9.74) |  |
| Non-insured | 238(9.55) | 46(6.17) | 192(11.00) |  |
| Married | 1020(40.93) | 228(30.56) | 792(45.36) | χ2(1) = 47.34, P < 0.001 |
| Urban residents | 1883(75.56) | 646(86.60) | 1237(70.85) | χ2(1) = 70.19, P < 0.001 |
| Diagnoses |  |  |  | χ2(4) = 151.42, P < 0.001 |
| Depressive disorder | 234(9.39) | 35(4.69) | 199(11.4) |  |
| Bipolar disorder | 420(16.85) | 43(5.76) | 377(21.59) |  |
| Schizophrenia and related disorders | 1375(55.18) | 517(69.30) | 858(49.14) |  |
| Substance use disorder | 132(5.30) | 30(4.02) | 102(5.84) |  |
| Other mental disorders | 331(13.28) | 121(16.22) | 210(12.03) |  |
| Count of comorbidities^#^ |  |  |  | χ2(2) = 153.61, P < 0.001 |
| None | 645(25.88) | 82(10.99) | 563(32.25) |  |
| 1 | 568(22.79) | 154(20.64) | 414(23.71) |  |
| >1 | 1279(51.32) | 510(68.36) | 769(44.04) |  |
| ECT | 451(18.10) | 41(5.50) | 410(23.48) | χ2(1) = 114.08, P < 0.001 |
| Previous hospitalization |  |  |  | χ2(3) = 759.29, P < 0.001 |
| None | 1233(49.48) | 122(16.35) | 1111(63.63) |  |
| 1 time | 430(17.26) | 92(12.33) | 338(19.36) |  |
| 2 times | 421(16.89) | 226(30.29) | 195(11.17) |  |
| >3 times | 408(16.37) | 306(41.02) | 102(5.84) |  |
| Admission sources |  |  |  | χ2(2) = 136.17, P < 0.001 |
| Emergency room | 591(23.72) | 70(9.38) | 521(29.84) |  |
| Outpatient | 1848(74.16) | 670(89.81) | 1178(67.47) |  |
| Other | 53(2.13) | 6(0.80) | 47(2.69) |  |
| Length of stay (mean, SD) | 79.83(63.43) | 101.33(50.48) | 70.65(66.13) | z = -15.17, P < 0.001 |
| Hospital info |  |  |  |  |
| Tertiary hospitals vs secondary hospitals | 2135(85.67) | 684(91.69) | 1451(83.1) | χ2(1) = 31.39, P < 0.001 |
| Urban hospital vs rural hospitals | 1084(43.5) | 146(19.57) | 938(53.72) | χ2(1) = 248.04, P < 0.001 |

| 4. Results of the 3 primary models and the sensitivity analyses | | | |
| --- | --- | --- | --- |
| Supplementary table 5. Logistic regression models of psychiatric readmissions in 30-days | | | |
|  | Full model | Sensitivity analyses | |
|  |  | Schizophrenia model | Affective disorder model |
| Male (ref. female) | 0.95(0.81,1.11) | 0.87(0.7,1.07) | 1.2(0.87,1.64) |
| Age (ref. (0-40]) |  |  |  |
| (40,65] | 0.84(0.68,1.04) | 1.16(0.87,1.55) | 0.51(0.34,0.77)** |
| >65 | 0.92(0.69,1.21) | 1.21(0.82,1.79) | 0.39(0.21,0.72)** |
| Insurance type (ref. no insurance) | | | |
| Urban employee | 1.19(0.88,1.6) | 1.14(0.74,1.77) | 1.12(0.68,1.85) |
| Urban resident | 0.75(0.51,1.09) | 0.99(0.59,1.68) | 0.74(0.35,1.53) |
| New rural cooperative | 1.12(0.74,1.71) | 1.31(0.72,2.39) | 0.99(0.48,2.04) |
| Other insurances | 1.03(0.71,1.50) | 1.1(0.63,1.90) | 0.96(0.52,1.78) |
| Married (ref. not married) | 0.86(0.72,1.02) | 0.8(0.63,1.02) | 0.93(0.65,1.34) |
| Urban residents (ref. Suburb) | 1.4(1.15,1.70)** | 1.26(0.96,1.65) | 1.3(0.91,1.86) |
| Diagnosis (ref. depressive disorders) | | | |
| Bipolar disorders | 1.1(0.79,1.54) | NA | NA |
| Schizophrenia and related disorders | 1.44(1.06,1.94)* | NA | NA |
| Substance use disorders | 0.64(0.4,1.04) | NA | NA |
| Other mental disorders | 1.3(0.93,1.81) | NA | NA |
| Count of comorbidities (ref. none) | | | |
| 1 | 1.67(1.34,2.09)** | 1.68(1.25,2.25)* | 2.18(1.45,3.28)** |
| >1 | 2.17(1.75,2.70)** | 2.38(1.8,3.15)** | 2.52(1.61,3.94)** |
| Use of ECT (ref. no ECT) | 0.72(0.56,0.91)* | 0.55(0.39,0.77)** | 0.87(0.6,1.26) |
| Psychiatric hospitalizations 1 year prior to index admission (ref. none) | | | |
| 1 | 1.91(1.55,2.36)** | 1.86(1.41,2.45)** | 1.81(1.13,2.91)* |
| 2 | 4.87(3.8,6.24)** | 4.78(3.47,6.58)** | 3.16(1.62,6.15)* |
| ≥3 times | 5.12(3.87,6.78)** | 3.66(2.56,5.24)** | 8.57(3.61,20.36)** |
| Admission sources (ref. emergency room) | |  |  |
| Outpatient | 1.13(0.92,1.39) | 1.38(1.004,1.9)* | 0.85(0.60,1.20) |
| Other | 0.53(0.31,0.90)** | 0.73(0.38,1.41) | 0.23(0.05,1.14) |
| Length of stay (ref. ≤20 days) |  |  |  |
| (20,30] | 0.45(0.33,0.62)** | 0.39(0.21,0.70)* | 0.41(0.26,0.65)** |
| (30,40] | 0.46(0.32,0.66)** | 0.44(0.24,0.79)* | 0.4(0.23,0.68)* |
| (40,60] | 0.53(0.38,0.74)** | 0.46(0.28,0.77)* | 0.4(0.23,0.70)* |
| (60,100] | 1.6(1.21,2.13)** | 1.76(1.15,2.69)* | 0.62(0.33,1.14)** |
| >100 | 3.27(2.46,4.35)* | 3.49(2.3,5.30)** | 2.71(1.45,5.08)* |
| Hospital characteristics |  |  |  |
| Tertiary hospital vs secondary hospitals | 2.61(2.04,3.35)** | 5.08(3.6,7.16)** | 1.09(0.6,2.01) |
| Urban hospitals vs rural hospitals | 0.54(0.44,0.66)** | 0.59(0.44,0.79)** | 0.46(0.31,0.68)** |
| Notes: * p<0.05; ** p<0.001; Number in the Table: Odds ratio (95% confidence interval). | | | |

| Supplementary table 6. Logistic regression models of psychiatric readmissions in 365-days | | | |
| --- | --- | --- | --- |
|  | Full model | Sensitivity analyses | |
|  |  | Schizophrenia model | Affective disorder model |
| Male (ref. female) | 1(0.89,1.12) | 0.92(0.76,1.1) | 1.02(0.85,1.23) |
| Age (ref. (0-40]) |  |  |  |
| (40,65] | 0.8(0.69,0.92)** | 0.87(0.70,1.08) | 0.85(0.67,1.09) |
| >65 | 0.69(0.55,0.87)** | 0.73(0.50,1.07) | 0.55(0.38,0.80)* |
| Insurance type (ref. no insurance) | |  |  |
| Urban employee | 1.42(1.17,1.72)** | 1.45(1.07,1.96)* | 1.39(1.03,1.86)* |
| Urban resident | 1.22(0.96,1.56) | 1.28(0.88,1.86) | 1.35(0.92,2.00) |
| New rural cooperative | 0.99(0.75,1.31) | 1.18(0.76,1.83) | 0.95(0.62,1.47) |
| Other insurances | 1.11(0.87,1.41) | 1.22(0.81,1.82) | 1.12(0.78,1.60) |
| Married (ref. not married) | 0.86(0.76,0.99)* | 0.72(0.59,0.88)* | 0.88(0.71,1.10) |
| Urban residents (ref. Suburb) | 1.41(1.23,1.61)** | 1.34(1.08,1.66)* | 1.38(1.12,1.70)* |
| Diagnosis (ref. depressive disorders) | |  |  |
| Bipolar disorders | 1.35(1.11,1.65)* | NA | NA |
| Schizophrenia and related disorders | 1.35(1.12,1.63)* | NA | NA |
| Substance use disorders | 1.14(0.85,1.53) | NA | NA |
| Other mental disorders | 1.18(0.94,1.47) | NA | NA |
| Count of comorbidities (ref. none) | |  |  |
| 1 | 1.62(1.39,1.89)** | 1.74(1.38,2.18)** | 1.73(1.36,2.19)** |
| >1 | 2.63(2.25,3.07)** | 2.97(2.35,3.76)** | 2.46(1.91,3.18)** |
| Use of ECT (ref. no ECT) | 0.91(0.79,1.05)** | 0.75(0.59,0.94)** | 0.97(0.79,1.19) |
| Psychiatric hospitalizations 1 year prior to index admission (ref. none) | | | |
| 1 | 1.96(1.66,2.31)** | 1.92(1.52,2.44)** | 1.89(1.41,2.53)** |
| 2 | 6.19(4.76,8.05)** | 7.15(5.01,10.20)** | 2.91(1.67,5.06)** |
| ≥3 times | 15.84(10.58,23.72)** | 15.16(8.87,25.92)** | 18.82(5.47,64.79)** |
| Admission sources (ref. emergency room) | |  |  |
| Outpatient | 1.07(0.94,1.22) | 1.14(0.92,1.42) | 1.04(0.85,1.26) |
| Other | 0.85(0.57,1.27) | 1.26(0.73,2.15) | 0.79(0.32,1.91) |
| Length of stay (ref. ≤20 days) |  |  |  |
| (20,30] | 0.87(0.73,1.03) | 0.83(0.59,1.17) | 0.89(0.69,1.13) |
| (30,40] | 0.8(0.66,0.98)* | 0.8(0.56,1.15) | 0.8(0.60,1.06) |
| (40,60] | 0.89(0.73,1.08) | 0.87(0.62,1.21) | 0.86(0.64,1.17) |
| (60,100] | 1.39(1.13,1.72)* | 1.59(1.13,2.22)* | 0.87(0.59,1.28) |
| >100 | 2.29(1.82,2.89)** | 2.71(1.92,3.82)** | 2.33(1.38,3.94)* |
| Hospital characteristics |  |  |  |
| Tertiary hospitals vs secondary hospitals | 1.38(1.14,1.67)* | 2.57(1.94,3.42)** | 0.88(0.60,1.31) |
| Urban hospitals vs rural hospitals | 0.75(0.65,0.87)** | 0.95(0.75,1.19) | 0.63(0.50,0.81)** |
| Notes: * p<0.05; ** p<0.001; Number in the Table: Odds ratio (95% confidence interval). | | | |

| Supplementary table 7. Logistic regression models of frequent psychiatric readmissions in a year | | | |
| --- | --- | --- | --- |
|  | Full model | Sensitivity analyses | |
|  |  | Schizophrenia model | Affective disorder model |
| Male (ref. female) | 1.3(1.04,1.64)* | 1.43(1.07,1.90)* | 1.05(0.60,1.85) |
| Age (ref. (0-40]) |  |  |  |
| (40,65] | 1.06(0.77,1.46) | 1.43(0.93,2.20) | 0.6(0.29,1.26) |
| >65 | 1.26(0.84,1.89) | 1.45(0.85,2.48) | 0.55(0.20,1.50) |
| Insurance type (ref. no insurance) | |  |  |
| Urban employee | 1.69(0.96,2.96) | 2.39(1.00,5.73) | 0.71(0.26,1.93) |
| Urban resident | 1.2(0.61,2.34) | 2.18(0.80,5.95) | 0.75(0.21,2.73) |
| New rural cooperative | 1.3(0.53,3.16) | 2.66(0.73,9.74) | 0.67(0.14,3.23) |
| Other insurances | 0.92(0.47,1.81) | 1.28(0.47,3.49) | 0.62(0.18,2.17) |
| Married (ref. not married) | 0.89(0.68,1.15) | 0.87(0.62,1.23) | 0.85(0.45,1.60) |
| Urban residents (ref. Suburb) | 1.45(1.07,1.95)* | 1.69(1.12,2.55)* | 1.28(0.67,2.44) |
| Diagnosis (ref. depressive disorders) | |  |  |
| Bipolar disorders | 0.47(0.27,0.82)* | NA | NA |
| Schizophrenia and related disorders | 0.78(0.49,1.26) | NA | NA |
| Substance use disorders | 0.81(0.43,1.54) | NA | NA |
| Other mental disorders | 1.2(0.72,2.03) | NA | NA |
| Count of comorbidities (ref. none) | |  |  |
| 1 | 1.59(1.11,2.27)* | 1.53(0.99,2.37) | 1.33(0.54,3.24) |
| >1 | 1.95(1.40,2.71)** | 1.66(1.11,2.47)* | 4.73(2.10,10.65)** |
| Use of ECT (ref. no ECT) | 0.6(0.40,0.91)* | 0.47(0.27,0.81)* | 0.95(0.49,1.85) |
| Psychiatric hospitalizations 1 year prior to index admission (ref. none) | | | |
| 1 | 1.66(1.19,2.32)* | 1.8(1.12,2.89)* | 1.63(0.75,3.56) |
| 2 | 4.79(3.45,6.66)** | 5.17(3.30,8.11)** | 3.9(1.58,9.61)* |
| ≥3 times | 10.02(7.05,14.24)** | 11.15(6.91,17.99)** | 10.9(3.97,29.89)** |
| Admission sources (ref. emergency room) | |  |  |
| Outpatient | 1.29(0.91,1.82) | 0.87(0.52,1.45) | 1.61(0.84,3.07) |
| Other | 0.93(0.32,2.75) | 0.4(0.08,1.84) | 3.28(0.27,40.56) |
| Length of stay (ref. ≤20 days) |  |  |  |
| (20,30] | 0.62(0.36,1.08) | 1.03(0.39,2.73) | 0.31(0.12,0.78)* |
| (30,40] | 0.84(0.47,1.49) | 1(0.38,2.67) | 0.52(0.20,1.37) |
| (40,60] | 1.3(0.79,2.14) | 1.29(0.54,3.04) | 0.81(0.34,1.96) |
| (60,100] | 2.48(1.56,3.95)** | 2.77(1.28,5.99)* | 1.12(0.41,3.03) |
| >100 | 1.26(0.79,2.01) | 1.32(0.62,2.82) | 0.92(0.31,2.70) |
| Hospital characteristics |  |  |  |
| Tertiary hospitals vs secondary hospitals | 2.3(1.51,3.51)** | 3.24(1.68,6.23)** | 5.01(1.19,21.06)* |
| Urban hospitals vs rural hospitals | 0.86(0.62,1.19) | 0.8(0.49,1.30) | 0.68(0.33,1.38) |
| Notes: * p<0.05; ** p<0.001; Number in the Table: Odds ratio (95% confidence interval). | | | |

5. Results for post-regression diagnostics of the primary and sensitivity analyses models

| Supplementary table 8. Model specification and regression Diagnostics | | | | | | | | | |
| --- | --- | --- | --- | --- | --- | --- | --- | --- | --- |
|  | 30-day readmission models | | | 365-day readmission models | | | Frequent readmission models | | |
|  | Full model | Schizophrenia model | Affective disorder model | Full model | Schizophrenia models | Affective disorder model | Full model | Schizophrenia models | Affective disorder model |
| No of index admissions | 7724 | 3348 | 2962 | 7734 | 3169 | 2938 | 2492 | 1375 | 663 |
| Incident rates (n (%)) | 1289(16.69) | 893(25.9) | 197(6.65) | 2492(33.79) | 1375(44.39) | 663(22.57) | 746(29.94) | 571(37.6) | 79(11.92) |
| Pseudo R2 | 0.35 | 0.39 | 0.16 | 0.23 | 0.31 | 0.09 | 0.31 | 0.31 | 0.24 |
| Mean VIF | 1.37 | 1.46 | 1.21 | 1.19 | 1.55 | 3.12 | 1.41 | 1.41 | 1.27 |
| C-statistics | 0.87 | 0.89 | 0.76 | 0.78 | 0.83 | 0.68 | 0.86 | 0.85 | 0.81 |
| Hosmer-Lemeshow test (Chi2(8)) | 35.17 | 41.45 | 11.12 | 16.63 | 15.51 | 12.81 | 12.61 | 6.02 | 9.17 |
| Hosmer-Lemeshow test (P value) | <0.001 | <0.001 | 0.19 | 0.03 | 0.05 | 0.12 | 0.13 | 0.64 | 0.33 |

For model discrimination and calibration, C-statistic was calculated from the receiver-operator characteristic curve to assess model discrimination, where a C-statistic of 0.50 or more indicates acceptable predictive power[1]. Hosmer-Lemeshow goodness-of-fit tests were applied and the calibration plots were generated for each model to assess model calibration[2].

It has been found that the results of the Hosmer-Lemeshow tests are prone to be significant when the sample was large[3], so we also included the calibration plots for the primary and sensitive analyses models.

6. Calibration plots for the primary and sensitivity analyses models[4]

| Supplementary table 9. Calibration plots for the primary and sensitivity analyses models | | | |
| --- | --- | --- | --- |
|  | Full models | Schizophrenia models | Affective disorder models |
| 30-day models | 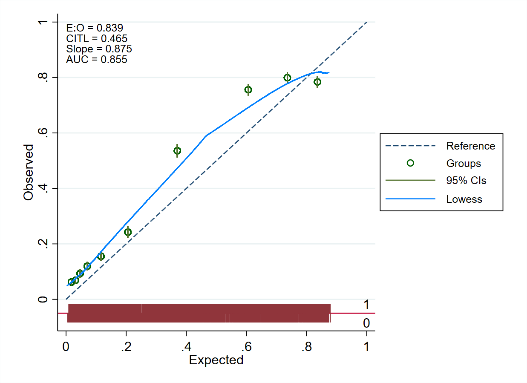 | 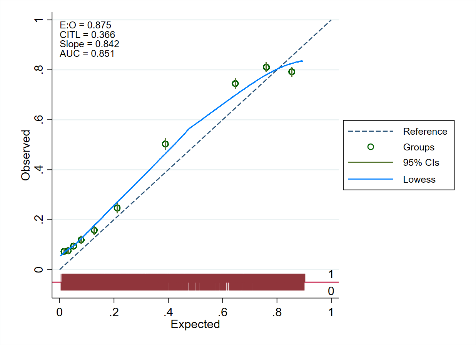 | 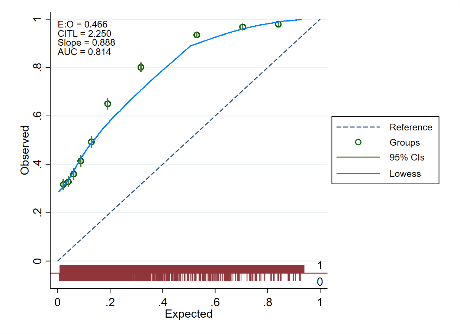 |
| 365-day readmission  models | 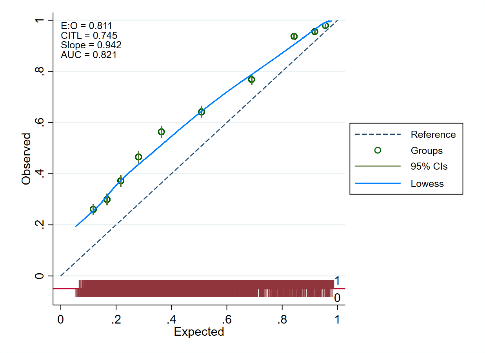 | 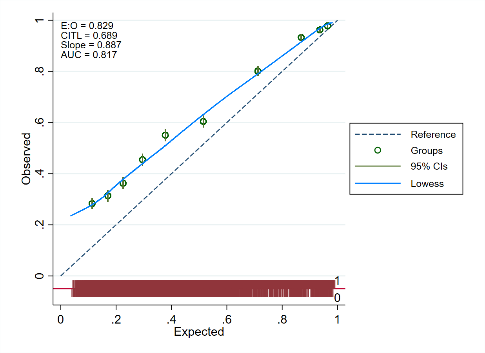 | 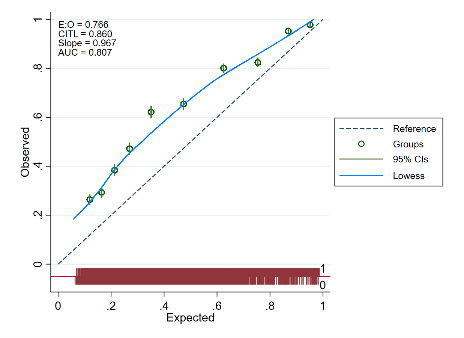 |
| Frequent readmission models | 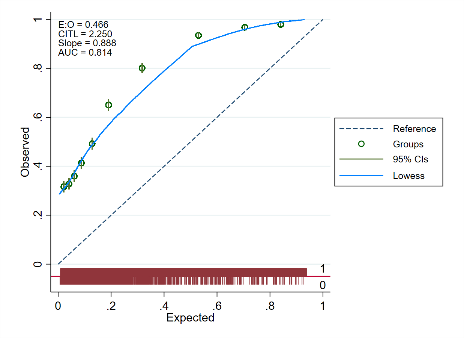 | 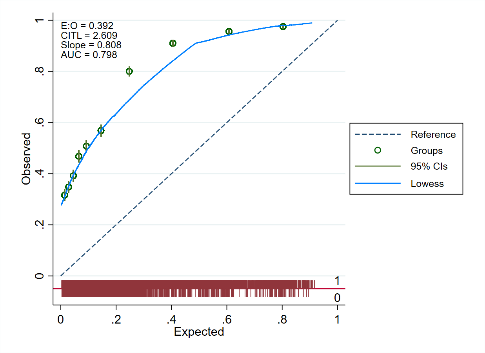 | 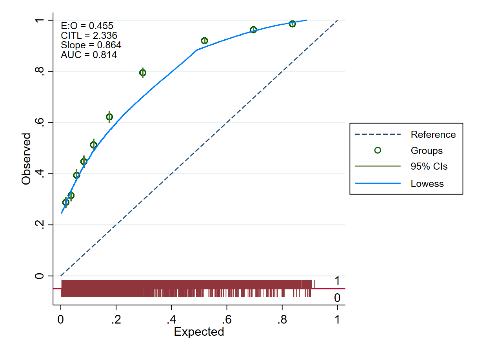 |

**References**

1. Matheny ME, Ohno-Machado L, Resnic FS: **Discrimination and calibration of mortality risk prediction models in interventional cardiology**. *J BIOMED INFORM* 2005, **38**(5):367-375.

2. Fenlon C, O Grady L, Doherty ML, Dunnion J: **A discussion of calibration techniques for evaluating binary and categorical predictive models**. *PREV VET MED* 2018, **149**:107-114.

3. Kramer AA, Zimmerman JE: **Assessing the calibration of mortality benchmarks in critical care: The Hosmer-Lemeshow test revisited***. *CRIT CARE MED* 2007, **35**(9):2052-2056.

4. Austin PC, Steyerberg EW: **Graphical assessment of internal and external calibration of logistic regression models by using loess smoothers**. *STAT MED* 2014, **33**(3):517-535.
